# Supplementary material for: PAM-flexible genome editing with an engineered chimeric Cas9
Source: Nat Commun. 2023 Oct 4;14:6175. doi: 10.1038/s41467-023-41829-y (PMC10550912; doi:10.1038/s41467-023-41829-y)
Supplement: Supplementary file 2 — Supplementary Information [file 41467_2023_41829_MOESM2_ESM.pdf]

<u>**Supplementary Information**</u>

PAM-Flexible Genome Editing with an Engineered Chimeric Cas9

Lin Zhao,[1,*] Sabrina R.T. Koseki,[1,*] Rachel A. Silverstein,[2-4] Nadia Amrani,[5] Christina Peng,[6] Christian Kramme,[7] Natasha Savic,[6] Martin Pacesa,[8] Tomás C. Rodríguez,[6] Teodora Stan,[1] Emma Tysinger,[1] Lauren Hong,[1] Vivian Yudistyra,[1] Manvitha R. Ponnapati,[2] Joseph M. Jacobson,[2] George M. Church,[7] Noah Jakimo,[9] Ray Truant,[6] Martin Jinek,[8] Benjamin P. Kleinstiver,[2,3,10] Erik J. Sontheimer,[5] Pranam Chatterjee[1,11,†]

1. Department of Biomedical Engineering, Duke University
2. Center for Genomic Medicine, Massachusetts General Hospital, Boston, MA, USA
3. Department of Pathology, Massachusetts General Hospital, Boston, MA, USA
4. Biological and Biomedical Sciences Program, Harvard University, Boston, MA, USA
5. RNA Therapeutics Institute, University of Massachusetts Medical School
6. Department of Biochemistry and Biomedical Sciences, McMaster University
7. Wyss Institute for Biologically Inspired Engineering, Harvard University
8. Department of Biochemistry, University of Zurich
9. Media Lab, Massachusetts Institute of Technology
10. Department of Pathology, Harvard Medical School, Boston, MA, USA
11. Department of Computer Science, Duke University

[†]Corresponding author: pranam.chatterjee@duke.edu

Supplementary Figures

Supplementary Figure 1. Gating strategy for PAM-SCANR FACS analysis.
Supplementary Figure 2. PAM characterization of SpRYc via endogenous base editing.
Supplementary Figure 3. GUIDE-Seq data including counts at each detected off-target for each nuclease tested.
Supplementary Figure 4. GUIDE-Seq data represented graphically of detected off-target read counts for each nuclease tested.
Supplementary Figure 5. Efficiency bar graph of mismatch tolerance assay on genomic targets.
Supplementary Figure 6. Sequencing of select SpRYc sgRNA DNA constructs from genomic DNA extracts to evaluate self-targeting.

Supplementary Table 1. sgRNA sequences used in Figures 2A-B.
Supplementary Table 2. sgRNA Sequences used in Supplementary Figure 2.
Supplementary Table 3. Sequences used in study.

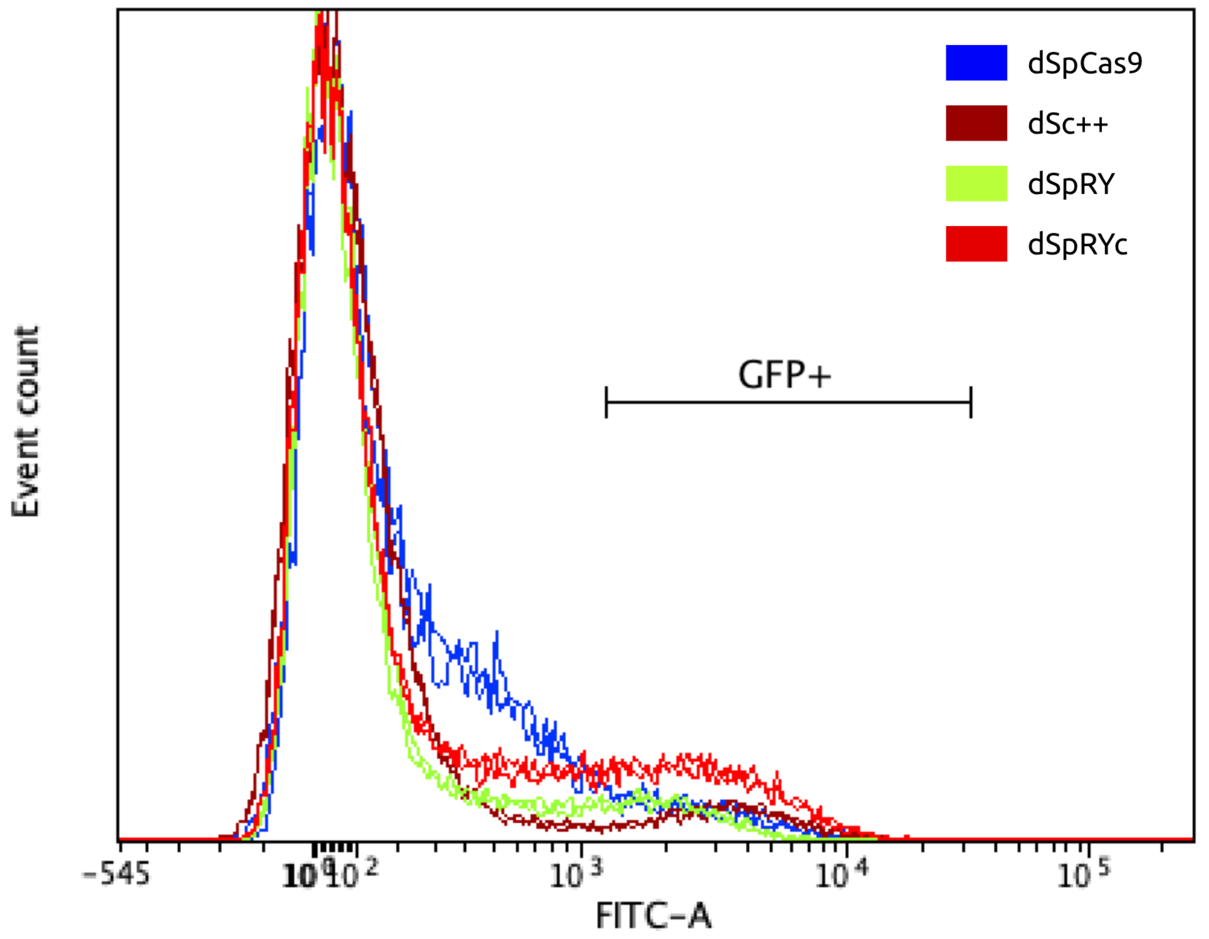

Supplementary Figure 1. 10,000 gated events for data analysis based on default FSC/SSC parameters for *E. coli*. The GFP+ gate was established both by a "no dCas9" negative control and a "dSpCas9" positive control.

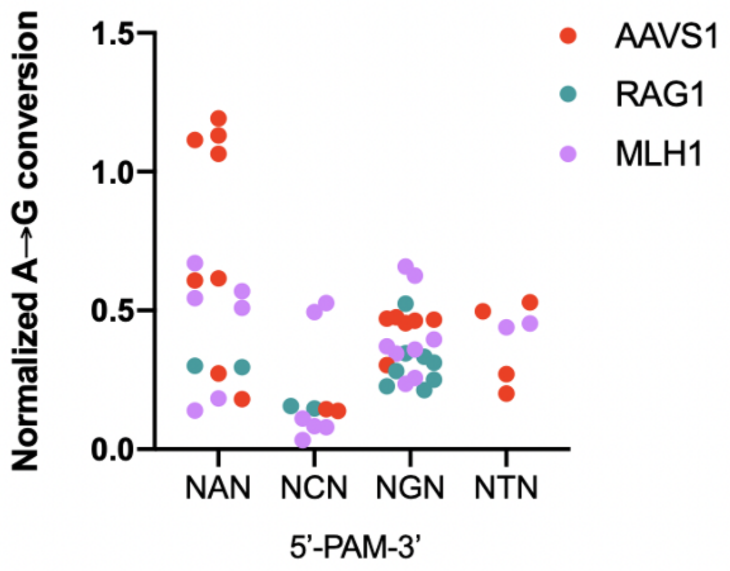

Supplementary Figure 2. PAM characterization of SpRYc via endogenous base editing. Dot plot of on-target normalized A→G modification rates on various gene targets for indicated PAM, as assayed by Beat (https://hanlab.cc/beat/) following PCR amplification of indicated genomic loci. All samples were performed in independent transfection replicates. Editing efficiency is indicated at various gene targets for indicated PAM, normalized with SpCas9-ABE8e for NGG PAM.

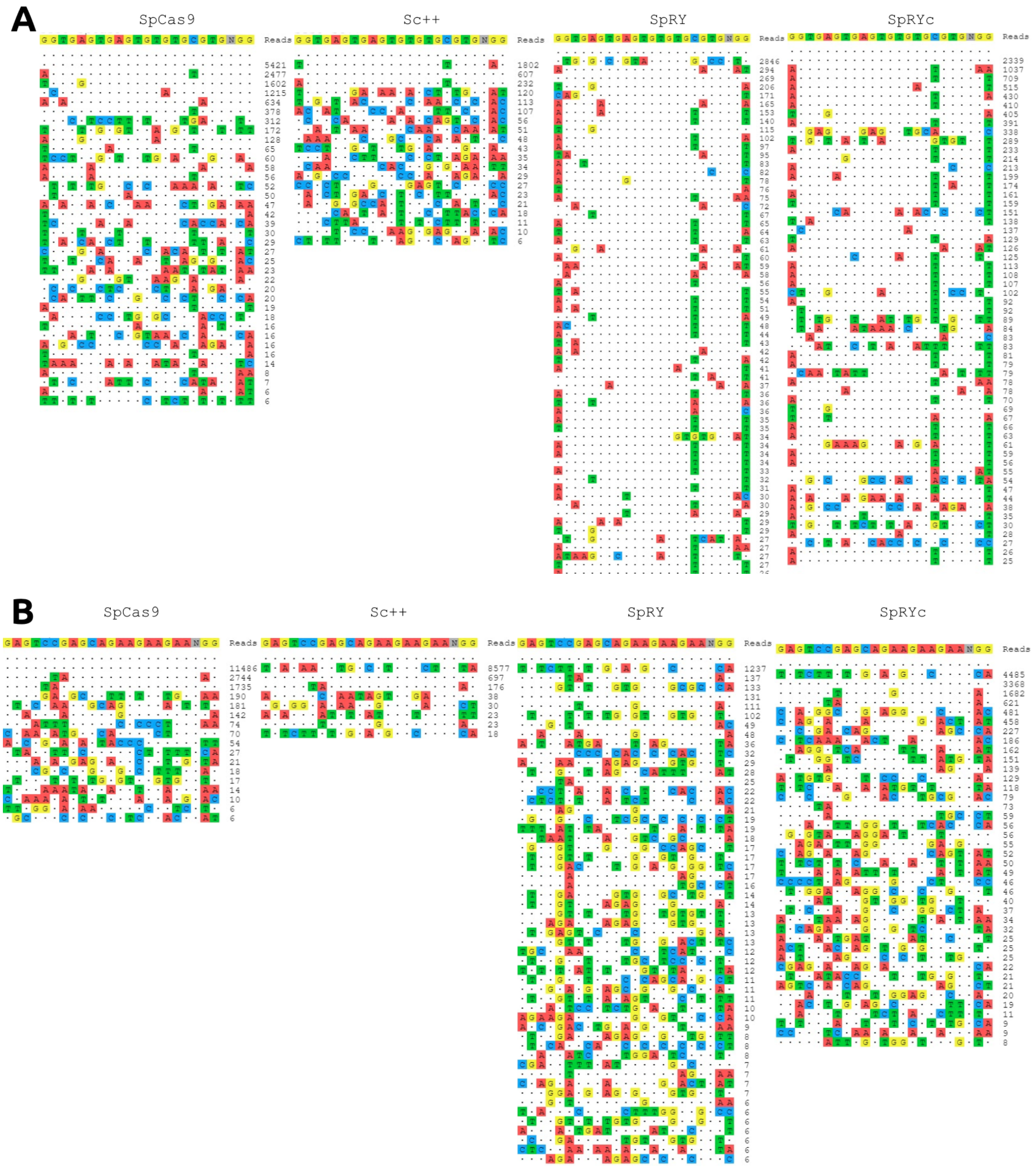

Supplementary Figure 3. For each Cas9, the on-target sequence is shown at the top with PAM in bold and with mismatches to the on-target site shown in color. GUIDE-Seq peak scores are shown to the right of each site. Data is shown for SpCas9, Sc++, SpRY, and SpRYc for the **A)** *VEGFA* site and the **B)** *EMX1* site.

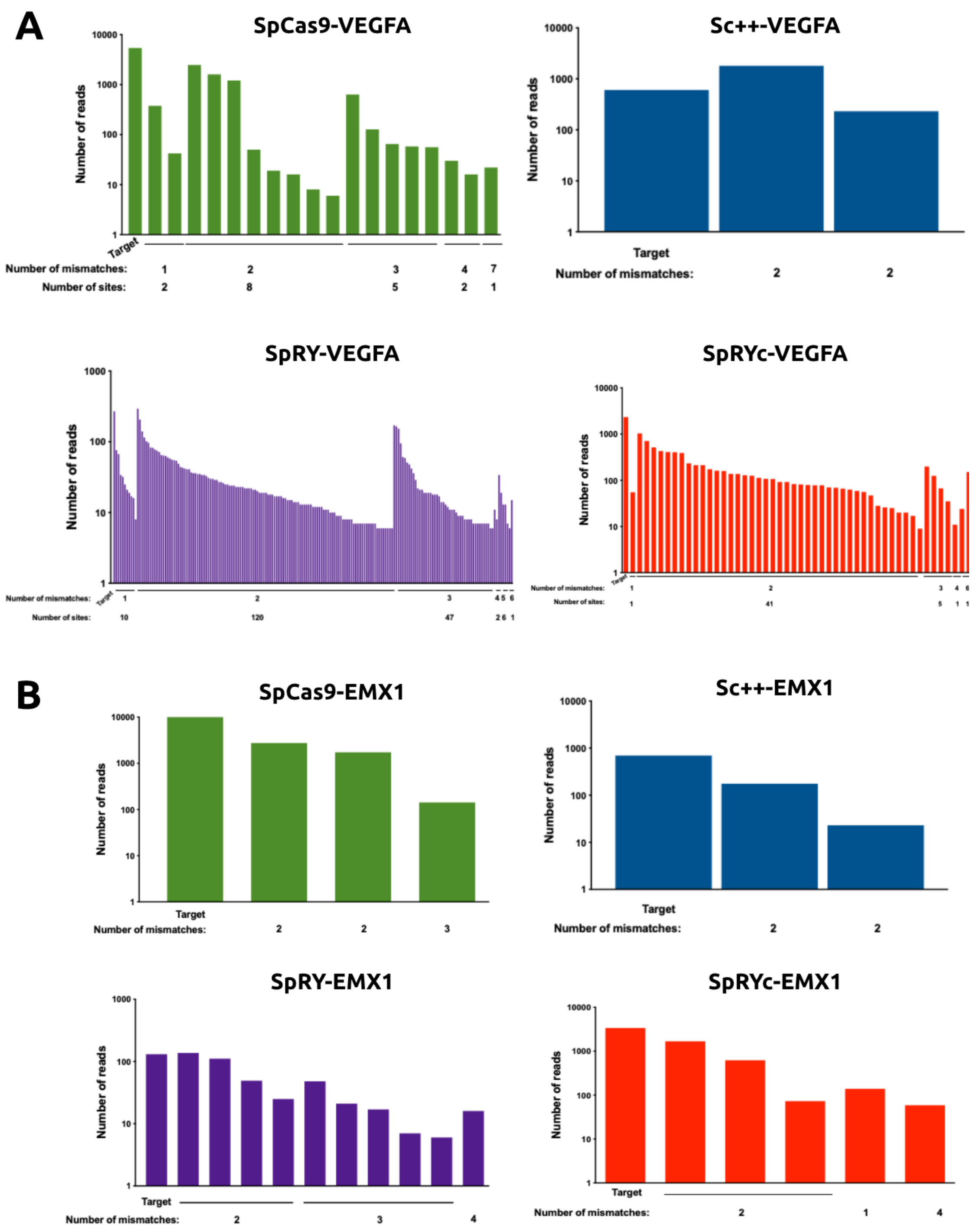

Supplementary Figure 4. Numbers of independent GUIDE-Seq reads for on- and off-target sites for all combinations of Cas9s (SpCas9, Sc++, SpRY, and SpRYc) and target sites **A)** *VEGFA* and **B)** *EMX1*, binned by the number of mismatches (≤6 mismatches) with the corresponding Cas9 and target site. The total number of sites in each bin are also shown.

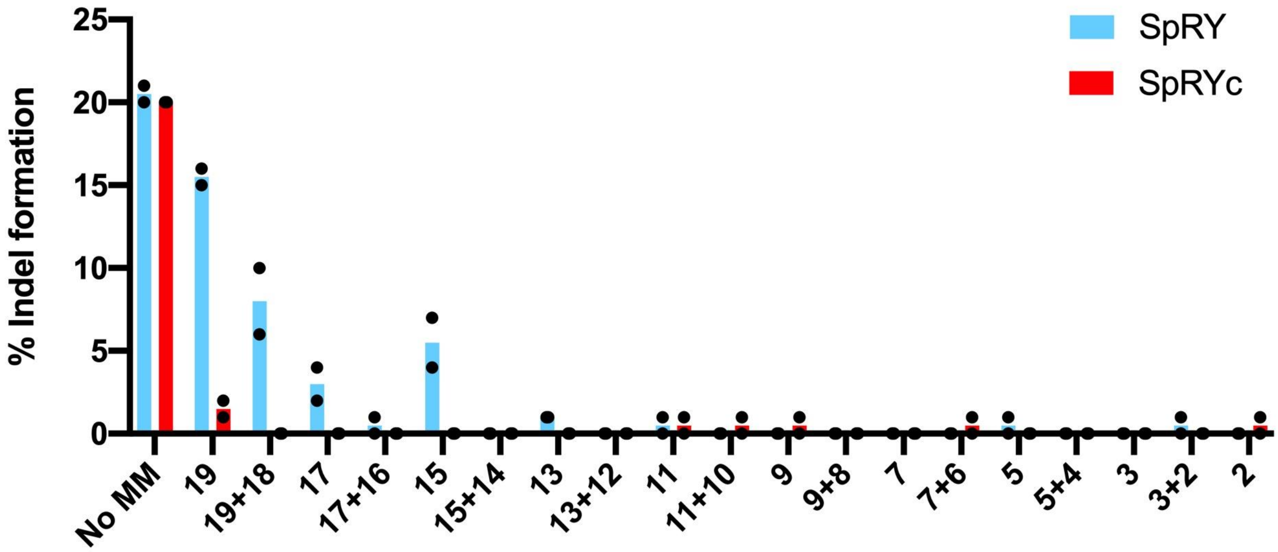

Supplementary Figure 5. Efficiency heatmap of mismatch tolerance assay on genomic targets. Quantified indel frequencies are exhibited for each labeled single or double mismatch (number of bases 5' upstream of the PAM) in the sgRNA sequence for the indicated Cas9 variant and indicated PAM sequence. All samples were performed in independent transfection replicates and the mean of the quantified indel formation values was calculated.

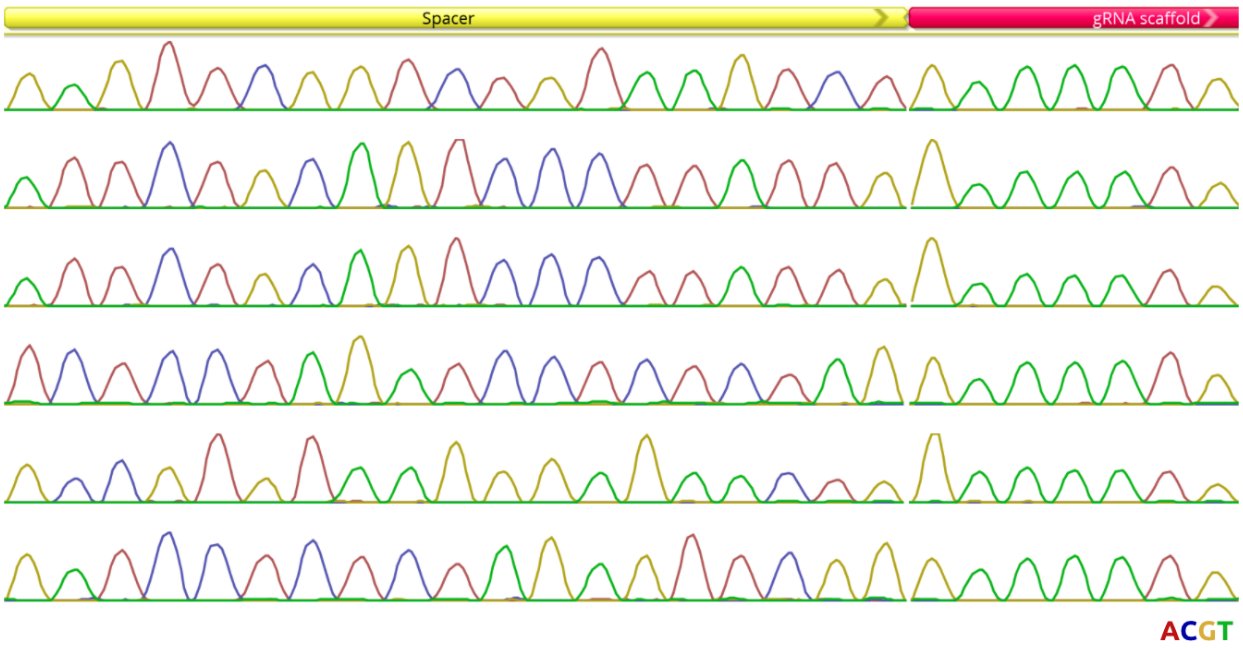

Supplementary Figure 6. Sequencing of select SpRYc sgRNA DNA constructs from genomic DNA extracts to evaluate self-targeting. HEK293T cells were transfected with SpRYc nuclease and indicated sgRNA construct.

Supplementary Table 1. sgRNA sequences used in Figures 2A-B.

sgRNA sequence	Gene	PAM	PAM Flank
AACAGACATGGACCATCAGG	DNMT1	AAACATTA	AA
CCAAGGCCACAAACACCATG	DNMT1	TACCACAC	AC
GAGCCAAATTCACCGAGCAG	DNMT1	GAGTGAGG	AG
CACAAACACCATGTACCACA	DNMT1	CATGTGAA	AT
TGTACCACACATGTGAACGG	DNMT1	ACAGATTG	CA
GAGCAGGAGTGAGGGAAACG	DNMT1	GCCCCAGG	CC
TCCCAGCTCGTAGTGCACCA	ZSCAN2	GCGGACCC	CG
ATGACCAGAATGGAGCCCCG	ZSCAN2	GCTGAAGC	CT
AACACCATGTACCACACATG	DNMT1	TGAACGGA	GA
GGCCGAGATTGGGTGTTCAG	PVALB	GGCAGAGA	GC
TTAACAGCTGACCCAATAAG	DNMT1	TGGCAGAG	GG
TGTGAACGGACAGATTGACA	DNMT1	TGTTAAAA	GT
TGAACGGACAGATTGACATG	DNMT1	TTAAAAAC	TA
GACTGAACACTCCTCAAACG	DNMT1	GTCCCCAG	TC
GTTAACAGCTGACCCAATAA	DNMT1	GTGGCAGA	TG
GTGAACGGACAGATTGACAT	DNMT1	GTTAAAAA	TT

Supplementary Table 2. sgRNA Sequences used in Supplementary Figure 2.

sgRNA Sequence	Gene	PAM	PAM Flank
GGTGACCCGAATCCACAGGA	AAVS1	GAACGGGG	AA
GGACAGATAAAAGTACCCAG	AAVS1	AACCAGAG	AC
GATAAAAGTACCCAGAACCA	AAVS1	GAGCCACA	AG
CGAATCCACAGGAGAACGGG	AAVS1	GTGTCCAG	TG
GGACCACCTTATATTCCCAG	AAVS1	GGCCGGTT	GC
AGGTACCTGAGAACAATGAA	RAG1	AACAAGTC	AC
GGTACCTGAGAACAATGAAA	RAG1	ACAAGTCA	CA
GCTGAGGTACCTGAGAACAA	RAG1	TGAAAACA	GA
GGGGCAGAACTGAGTCCCAA	RAG1	GGTGGGTG	GT
GTTGTCTTAATGGTACCGTT	MLH1	AACTAAGT	AC
GAGCGGTAAAGAAACACACG	MLH1	GTCTGCGG	TC
TAAGGGCTACGACTTAACGG	MLH1	GCCGCGTC	CC
CCGGGCAGAGGCATGTACAG	MLH1	CGCATGCC	GC
ATATTCCTCCACTTACACTC	MLH1	CAAACAAC	AA
GTACCGTTAACTAAGTAAGG	MLH1	AAGCCACT	AG
TGGTACCGTTAACTAAGTAA	MLH1	GGAAGCCA	GA
GGGACCACCTTATATTCCCA	AAVS1	GGGCCGGT	GG
GGGAATATAAGGTGGTCCCA	AAVS1	GCTCGGGG	CT
AAGGAGTGAGAGGTGACCCG	AAVS1	AATCCACA	AT
GGTGGAGGGGACAGATAAAA	AAVS1	GTACCCAG	TA
ACAGACTAGAGAGGTAAGGG	AAVS1	GGGTAGGG	GG
TTATTTATAAGATACATCAG	RAG1	TGGGATAT	GG
GCTTAAGATGGGGAGAAAAA	MLH1	CCTTTTTT	CT
GCAGACCGTGTGTTTCTTTA	MLH1	CCGCTCTC	CG
GAGAGCGGTAAAGAAACACA	MLH1	CGGTCTGC	GG
TTATTTATAAGATACATCAGT	RAG1	GGGATATT	GG
TGGCACGTCAGGGAACCCGG	MLH1	CGGCCTCC	GG

sgRNA Sequence	Gene		PAM Flank
GGGACCACCTTATATTCCCA	AAVS1	GGGCCGGT	GG
TTATTTATAAGATACATCAGT	RAG1	GGGATATT	GG
TGGCACGTCAGGGAACCCGG	MLH1	CGGCCTCC	GG

Supplementary Figure 3. Sequences used in study.

Gene/Construct	Forward Primer	Reverse Primer
PAM-SCANR Library	AGATCCTTGGCGGCAAGAAA	CGCGGGAAACGGTCTGATAA
DNMT1	CCAGAATGCACAAAGTACTGCAC	GCCAAAGCCCGAGAGAGTGCC
PVALB	CTGGAAAGCCAATGCCTGAC	GGCAGCAAACTCCTTGTCCT
ZSCAN2	AGCCAGAGCTCCAGTCTGAT	CGGGACTTGACTCAGACCAC
AAVS1	CAACCCCAAAGTACCCCGTC	AAATGGGGGTGTGTCACCAG
RAG1	GGGGAGGCAAAGATGAATCAAA	AGAGGGTTTCCCCTCAAAGG
MLH1	AGCGGCCAGCTAATGCTAT	AAGAAACTCAAAATGAATTGTGCC
RTT Locus	AGTATGATGTTTGTTCCTTGTGTC	CAAGGAGCTTCCCAGGACTT
HTT	CCGCTCAGGTTCTGCTTTTA	GGCTGAGGCAGCAGCGGCTG

sgRNA	crRNA Sequence	PAM
HTT sgRNA	GCTGCTGCTGCTGCTGCTGG	AAGGACTT
RTT_C502T	TGCTCTCACCGGGAGGGGCT	CCCTCTCC
VEGFA_GuideSeq_sgRNA	GGTGAGTGAGTGTGTGCGTG	TGGGGTTG
EMX1_GuideSeq_sgRNA	GAGTCCGAGCAGAAGAAGAA	GGGCTCCC

SpRYc amino acid sequence:

MEKKYSIGLDIGTNSVGWAVITDDYKVPSKKFKVLGNTNRKSIKKNLMGALLFDSGETAEATRLKRTARRRYTRRKNRIRYLQEIFANEMAKLDDSFFQRLEE
SFLVEEDKKNERHPIFGNLADEVAYHRNYPTIYHLRKKLADSPEKADLRLIYLALAHIIKFRGHFLIEGKLNAENSDVAKLFYQLIQTYNQLFEESPLDEIEVDAK
GILSARLSKSKRLEKLIAVFPNEKKNGLFGNIIALALGLTPNFKSNFDLTEDAKLQLSKDTYDDDLDELLGQIGDQYADLFSAAKNLSDAILLSDILRSNSEVTKA
PLSASMVKRYDEHHQDLALLKTLVRQQFPEKYAEIFKDDTKNGYAGYVGADKKLRKRSGKLATEEEFYKFIKPILEKMDGAEELLAKLNRDDLLRKQRTFDN
GSIPHQIHLKELHAILRRQEEFYPFLKENREKIEKILTFRIPYYVGPLARGNSRFAWLTRKSEEAITPWNFEEVVDKGASAQSFIERMTNFDEQLPNKKVLPKH
SLLYEYFTVYNELTKVKYVTERMRKPEFLSGEQKKAIVDLLFKTNRKVTVKQLKEDYFKKIECFDSVEIIGVEDRFNASLGTYHDLLKIIKDKDFLDNEENEDIL
EDIVLTLTLFEDREMIEERLKTYAHLFDDKVMKQLKRRHYTGWGRLSRKMINGIRDKQSGKTILDFLKSDGFSNRNFMQLIHDDSLTFKEEIEKAQVSGQGDS
LHEQIADLAGSPAIKKGILQTVKIVDELVKVMGHKPENIVIEMARENQTTTKGLQQSRERKKRIEEGIKELESQILKENPVENTQLQNEKLYLYYLQNGRDMYV
DQELDINRLSDYDVDHIVPQSFIKDDSIDNKVLTRSVENRGKSDNVPSEEVVKKMKNYWRQLLNAKLITQRKFDNLTKAERGGLSEADKAGFIKRQLVETRQI
TKHVARILDSRMNTKRDKNDKPIREVKVITLKSKLVSDFRKDFQLYKVRDINNYHHAHDAYLNAVVGTALIKKYPKLESEFVYGDYKVYDVRKMIAKSEQEIGK
ATAKRFFYSNIMNFFKTEVKLANGEIRKRPLIETNGETGEVVWNKEKDFATVRKVLAMPQVNIVKKTEVQTGGFSKESIRPKRNSDKLIARKKDWDPKKYGG
FLWPTVAYSVLVVAKVEKGKSKKLKSVKELLGITIMERSSFEKNPIDFLEAKGYKEVKKDLIIKLPKYSLFELENGRKRMLASAKQLQKGNELALPSKYVNFLYL
ASHYEKLKGSPEDNEQKQLFVEQHKHYLDEIIEQISEFSKRVILADANLDKVLSAYNKHRDKPIREQAENIIHLFTLTRLGAPRAFKYFDTTIDPKQYRSTKEVL
DATLIHQSITGLYETRIDLSQLGGD

Synthetic RTT locus DNA sequence used in Figure 3A and 3B:

ACCCATGTATGATGACCCCACCCTGCCTGAAGGCTGGACATGGAAGCTTAAGCAAAGGAAATCTGGCCGCTCTGCTGGGAAGTATGATGTTTGTTCC
TTGTGTCTTTCTGTTTGTCCCCACAAGTCCCCAGGGAAAAGCCTTTTGCTCTAAAGTGGAGTTGATTGCGTACTTCGAAAAGGTAGGCGACACATCCC
TGGACCCTAATGATTTTGACTTCATGGTAACTGGGAGAGGGAGCCCCTCCCGGTGAGAGCAGAAACCACCTAAGAAGCCCAAATCTCCCAAAGCTCC
AGGAACTGGCAGAGGCCGGGGACGCCCCAAAGGGAGCGGCACCACGAGACCCAAGGCGGCCACGTCAGAGGGTGTGCAGGTGAAAAGGGTCCT
GGAGAAAAGTCCTGGGAAGCTCCTTGTCAAGATGCCTTTTCAAACTTCGCCAGGGGGCAAGGCTGAGGGGGGTGGGGCCACCACATCCACCCAGG
TCATGGTGATCAAACGCCCCGGCAGGAAGTGAAAAGCTGAGGCCGACCCTCAGGCCATTCCCAAGAAACGGGGCTGAAAGCCGGGGAGTGTGGT
GGCAGCCGCTGCCGCCGAGGCCAAAAAGAAAGCCGTGAAGGAGTCTTCTATCTGATCTGTGCAGGAGACCGTACTCCCCATCAAGAAGTGCAAGAC
CCGGGAGACGGTCAGCATCGAGGTCAAGGAA
