# Supplementary material for: PAM-flexible genome editing with an engineered chimeric Cas9
Source: Nat Commun. 2023 Oct 4;14:6175. doi: 10.1038/s41467-023-41829-y (PMC10550912; doi:10.1038/s41467-023-41829-y)
Supplement: Supplementary file 3 — Reporting Summary [file 41467_2023_41829_MOESM3_ESM.pdf]

nature research

Corresponding author(s): Pranam Chatterjee

Last updated by author(s): 09/18/2023

# Reporting Summary

Nature Research wishes to improve the reproducibility of the work that we publish. This form provides structure for consistency and transparency in reporting. For further information on Nature Research policies, see our Editorial Policies and the Editorial Policy Checklist.

## Statistics

For all statistical analyses, confirm that the following items are present in the figure legend, table legend, main text, or Methods section.

| n/a | Confirmed | |
|---|---|---|
| ☐ | ☒ | The exact sample size ($n$) for each experimental group/condition, given as a discrete number and unit of measurement |
| ☐ | ☒ | A statement on whether measurements were taken from distinct samples or whether the same sample was measured repeatedly |
| ☐ | ☒ | The statistical test(s) used AND whether they are one- or two-sided<br>*Only common tests should be described solely by name; describe more complex techniques in the Methods section.* |
| ☒ | ☐ | A description of all covariates tested |
| ☒ | ☐ | A description of any assumptions or corrections, such as tests of normality and adjustment for multiple comparisons |
| ☐ | ☒ | A full description of the statistical parameters including central tendency (e.g. means) or other basic estimates (e.g. regression coefficient) AND variation (e.g. standard deviation) or associated estimates of uncertainty (e.g. confidence intervals) |
| ☐ | ☒ | For null hypothesis testing, the test statistic (e.g. $F$, $t$, $r$) with confidence intervals, effect sizes, degrees of freedom and $P$ value noted<br>*Give P values as exact values whenever suitable.* |
| ☒ | ☐ | For Bayesian analysis, information on the choice of priors and Markov chain Monte Carlo settings |
| ☒ | ☐ | For hierarchical and complex designs, identification of the appropriate level for tests and full reporting of outcomes |
| ☒ | ☐ | Estimates of effect sizes (e.g. Cohen's $d$, Pearson's $r$), indicating how they were calculated |

*Our web collection on statistics for biologists contains articles on many of the points above.*

## Software and code

Policy information about availability of computer code

| | |
|---|---|
| Data collection | Amplicons were enzymatically purified and submitted for Sanger sequencing or Amplicon-EZ NGS sequencing (Genewiz). Raw sequencer output (BCL) for GUIDE-Seq was demultiplexed and aligned to hg38 using GS-Preprocess (github.com/umasstr/GS-Preprocess). This software also constructed a reference of UMIs unique to each read and merged technical replicate BAM files. Off-target analysis of this input was performed using the GUIDEseq Bioconductor package |
| Data analysis | Sanger sequencing ab1 files were analyzed using the ICE web tool for batch analysis ice.synthego.com in comparison to an unedited control to calculate indel frequencies via the ICE-D score. Select samples were further verified using the TIDE algorithm tide.deskgen.com) to ascertain consistency of editing rates between replicates. NGS FASTQ files were analysed using a batch version of the software CRISPResso2 (https://github.com/pinellolab/CRISPResso2) . Base editing files were analyzed via the Based Editing Evaluation Program (BEEP) (https://github.com/mitmedialab/BEEP) in comparison to an unedited control. All samples were performed in independent duplicates or triplicates, as indicated. FCS files were analyzed using FCSalyzer https://sourceforge.net/projects/fcsalyzer/, and gating strategy is described in Supplementary Figure 1. PAM depletion was quantified following NGS of PCR-amplified undigested target DNA via the PAMDA software package: https://github.com/kleinstiverlab/HT-PAMDA. |

For manuscripts utilizing custom algorithms or software that are central to the research but not yet described in published literature, software must be made available to editors and reviewers. We strongly encourage code deposition in a community repository (e.g. GitHub). See the Nature Research guidelines for submitting code & software for further information.

## Data

Policy information about availability of data

All manuscripts must include a data availability statement. This statement should provide the following information, where applicable:

- Accession codes, unique identifiers, or web links for publicly available datasets
- A list of figures that have associated raw data
- A description of any restrictions on data availability

All data needed to evaluate the conclusions in the paper are present in the paper and supplementary tables. Sequence data that support the findings of this study are available via the NIH Sequence Read Archive via BioProject PRJNA1019291. Raw data underlying graphical figures are provided as Source Data. All additional data can be found at the following Zenodo repository: 10.5281/zenodo.8305744. The SpRYc-ABE8e (#208336) and SpRYc-BE4Max (#208340) plasmids have been deposited to Addgene.

# Field-specific reporting

Please select the one below that is the best fit for your research. If you are not sure, read the appropriate sections before making your selection.

☒ Life sciences ☐ Behavioural & social sciences ☐ Ecological, evolutionary & environmental sciences

For a reference copy of the document with all sections, see nature.com/documents/nr-reporting-summary-flat.pdf

# Life sciences study design

All studies must disclose on these points even when the disclosure is negative.

| | |
|---|---|
| Sample size | No sample size calculation was performed. For PAM analysis in human cells, we utilized a random set of sgRNA sequences to cover all PAM sequences for testing. For indel analysis, we tested 16sgRNAs, together directed to various genomic loci with diverse PAM sequences, collectively representing every base at each position in the PAM window. This information is present in the manuscript. |
| Data exclusions | No data was excluded from the study. All values shown represent all data collected in the study. |
| Replication | All samples were performed in independent biological duplicates (n=2) or triplicates (n=3), as indicated. All attempts at replication were successful. |
| Randomization | sgRNA sequences and PAMs were chosen randomly without any sequence bias. Samples were randomly allocated into experimental groups, if needed. |
| Blinding | Blinding is not relevant to this study, as specific sequences needed to be identified prior to experimental setup. |

# Reporting for specific materials, systems and methods

We require information from authors about some types of materials, experimental systems and methods used in many studies. Here, indicate whether each material, system or method listed is relevant to your study. If you are not sure if a list item applies to your research, read the appropriate section before selecting a response.

## Materials & experimental systems

| n/a | Involved in the study | |
|---|---|---|
| ☒ | ☐ | Antibodies |
| ☐ | ☒ | Eukaryotic cell lines |
| ☒ | ☐ | Palaeontology and archaeology |
| ☒ | ☐ | Animals and other organisms |
| ☒ | ☐ | Human research participants |
| ☒ | ☐ | Clinical data |
| ☒ | ☐ | Dual use research of concern |

## Methods

| n/a | Involved in the study | |
|---|---|---|
| ☒ | ☐ | ChIP-seq |
| ☐ | ☒ | Flow cytometry |
| ☒ | ☐ | MRI-based neuroimaging |

# Eukaryotic cell lines

Policy information about cell lines

| | |
|---|---|
| Cell line source(s) | 293T ATCC CRL-3216, TruHD-Q43Q17M (derived from ND30013) |
| Authentication | All genomic sequences tested in this study were PCR amplified and verified before conducting gene editing experiments. |
| Mycoplasma contamination | Cell lines were tested negative for mycoplasma contamination. |
| Commonly misidentified lines (See ICLAC register) | None used in this study. |

# Flow Cytometry

## Plots

Confirm that:

- [x] The axis labels state the marker and fluorochrome used (e.g. CD4-FITC).
- [x] The axis scales are clearly visible. Include numbers along axes only for bottom left plot of group (a 'group' is an analysis of identical markers).
- [x] All plots are contour plots with outliers or pseudocolor plots.
- [x] A numerical value for number of cells or percentage (with statistics) is provided.

## Methodology

| | |
|---|---|
| Sample preparation | Bacterial cells were grown to an OD of 0.2 overnight and were diluted to equivalent concentrations in PBS for analysis. |
| Instrument | BD FACSAria™ III: 4 laser, 12 color sorter. |
| Software | BDFACS Diva software was used to collect data. Open source FCSalyzer (https://sourceforge.net/projects/fcsalyzer/) was used to analyze Flow Cytometry data. |
| Cell population abundance | 10,000 gated events for data analysis were collected based on default FSC/SSC parameters for E. Coli. |
| Gating strategy | 10,000 gated events for data analysis based on default FSC/SSC parameters for E. coli. The GFP+ gate was established both by a "no Cas9" negative control and a "SpCas9" positive control. |

- [x] Tick this box to confirm that a figure exemplifying the gating strategy is provided in the Supplementary Information.
